# CMIP6 projections for global offshore wind and wave energy production (2015–2100)

**DOI:** 10.1038/s41598-023-45450-3

**Published:** 2023-10-23

**Authors:** Gabriel Ibarra-Berastegui, Jon Sáenz, Alain Ulazia, Aitor Sáenz-Aguirre, Ganix Esnaola

**Affiliations:** 1 Energy Engineering Department, University of the Basque Country (UPV/EHU), Plaza Ingeniero Torres Quevedo, 1, 48013 Bilbao, Spain; 2 Department of Physics, University of the Basque Country (UPV/EHU), Barrio Sarriena, 48940 Leioa, Spain; 3 Energy Engineering Department, University of the Basque Country (UPV/EHU), Otaola Hiribidea, 29, 20600 Eibar, Spain; 4 Energy Engineering Department, University of the Basque Country (UPV/EHU), Plaza Europa, 1, 20018 Donostia, Spain; 5 Plentziako Itsas Estazioa, University of the Basque Country (UPV/EHU), Areatza Hiribidea, 47, 48620 Plentzia, Spain

**Keywords:** Climate sciences, Renewable energy

## Abstract

Three-hourly CMIP6 projections have been used in conjuction with the CSIRO WaveWatchIII wave model to calculate the global trends in offshore wind and wave energy for the SSP585 and SSP126 scenarios until 2100. The results indicate that moderate yet significant changes are expected in the theoretical electricity generated from wind and waves at fewer than 10–15% of coastal locations. While this implies a generally stable outlook for the future, certain coastal regions with existing or planned wind farms may experience a slight reduction in production by 2100. Regarding wave energy, given its early stage of development, a more cautious approach is advisable, although a similar conclusion may be reached. Considering the decreasing installation costs on the horizon and accounting for both climatic scenarios, this provides a reliable context for most ongoing feasibility studies, technological developments, and offshore facility investments.

## Introduction

Following the latest IPCC report^1^, two reports by the International Energy Agency (*IEA*)^2^ and the International Renewable Energy Agency (*IRENA*)^3^ recently highlighted the need to increase the use of renewable energies to limit global warming to 1.5$^{\circ }$C by 2100. Therefore, global investment in renewables must be tripled to meet climate and development goals^4^. It is estimated that an annual increase by 329 GW/year of additional installed wind energy is needed over the coming decades to meet the 1.5 $^{\circ }$C target^3^. A significant part of this global effort^5^ will involve offshore wind facilities. For wave energy, an estimated 29500 TWh/year could be harnessed from ocean waves^6^. However, the installed capacity in wave farms accounts for only a small share (13.5 MW) of the global capacity of renewables, which is currently estimated at 2600 GW^6^. Additionally, while wind energy is currently a reliable source of energy, wave energy is still in its infancy in terms of technological development with low technology readiness levels (*TRL*) values^7–9^. The practical implementation of the above targets for both offshore wind and wave energy depends on the following aspects:

1. decisions made by national authorities and regulatory bodies on coastal and marine planning; and
2. the results of feasibility studies at candidate locations that may ensure private sector investors of a suitable return on the investments made.

A key aspect of any feasibility study is the accurate assessment of available local wind and wave resources. Other additional aspects like local and international legislation, stability, risks evaluation or even model specifications must also be incorporated into any feasibility assessment study^10^. Assuming a lifetime of several decades during which profitability must not be compromised, this applies not only to present-day conditions but also to their future evolution^11^. An additional aspect mentioned in the IPCC reports^12^ is the rise in sea levels, and this also needs to be considered in the feasibility analysis of any offshore wind or wave farm. Currently, the sea level is rising at a rate of 3.7 mm/year^12,13^, and no significant impact is expected on the energy performance of bottom-fixed, moored, or floating devices. However, this parameter needs to be incorporated into the general layout and planning of any offshore facility because auxiliary electric equipment is deployed at coastal sites that will have to be selected carefully.

Some types of wave farms need to be located on coastal boundaries^14^, but the rising sea level over the coming decades is expected to have only a negligible effect on their general performance^8,15^. Therefore, the rise in sea level will not be incorporated into this analysis, and the focus will be on the future evolution of the resource. The most recent estimations by climate models until 2100 have been used here to assess the global evolution of wind and waves and estimate changes in offshore energy production. Accordingly, the results from two CMIP6^16^ climate model simulations (EC-Earth3 and ACCESS-CM2) have been coupled with the WaveWatchIII (WWIII) wave model^17^ at the Australian CSIRO. The figures have recently been released^18^ and include three-hourly global ocean wind and wave data corresponding to the 1961-2100 period for the SSP585 and SSP126 scenarios^19,20^. Following the guidelines provided by referential studies^11^ and institutions in the definition of standard climate norms^21^, a 30-year period from 1985 to 2014 was selected to define present-day conditions.

The objective of this paper is to use CMIP6-WWIII monthly anomalies to estimate the global long-term trends in offshore wind and wave energy production for both climate scenarios for the 2100 horizon with respect to current conditions. The focus is the coastal areas where offshore facilities can be deployed, although future technological developments may extend their range.

## Main text

The evaluation of changes in offshore energy production will involve CMIP6 projections of global wind and wave resources combined with the estimated output of two standard devices, namely, a wind turbine (*WT*) and a wave energy converter (*WEC*). This allows for a referential assessment of the theoretical electricity generated by both wind and waves for each grid point where an offshore facility is technically feasible. The output for the selected *WT* has been derived from its capacity factor (*CF*), calculated by introducing global wind and wave values into a set of widely accepted semiempirical expressions. In the case of the selected *WEC*, the output has been estimated using the mathematical expressions provided by its designers.

Different types of *WT*s and a wide range of *WEC* designs^22^ will be deployed in offshore facilities until 2100. However, if comparable results are obtained across locations worldwide over the long timeframe analysed here, the energy generated by two standard devices must be analysed. Once their performance has been characterised, the conclusions may be transferred to any other *WT* or *WEC* design. This standardised approach provides the offshore renewable industry with a realistic assessment of how climate-driven changes may impact the sector over the coming decades, thus helping to reduce financial costs and risks, which are two of the challenges recently identified by stakeholders^23^. It may also help governments identify marine areas whose environmental protection on the one hand and national efforts to meet $CO_2$ emission targets on the other hand, can be guaranteed, together with profitability for the private sector. Any step forward along these lines will contribute to the more rapid development of the offshore renewable industry by speeding up licences and permits, currently a major barrier to its expansion^23^.

In the case of wave energy, the device selected for this study (see "Offshore wave energy: selected device, current energy production and global trends" section) can work either anchored to the bottom or floating whereby the same engineering limitations used for wind energy apply. For this reason, 2015-2100 trends have been calculated only in ocean areas with depths of less than 1000 m and less than 200 km offshore. Figure 2 shows the areas that meet these conditions for both types of offshore facilities.

### Offshore wind energy: selected device, current energy production and global trends

Regarding wind energy, a *WT* with a 5 MW power ranking ($P_R$, rated power) and a hub height of 90 m was selected^24^ because of its widespread use in similar studies^25^, including the authors’ analysis of this benchmark device in previous studies^26^. With a a rotor radius of about 63 m and a cut-in speed of 3 m/s, this 3-blade device can be installed in both, bottom-fixed and floating platforms.

Current electricity production and future trends have been derived for this device from its *CF*, which takes a given period as a reference (usually one year) and represents the ratio between the energy generated and the energy that could have been produced by a wind turbine if working at its $P_R$ during that period (Eq. 1)^27^. Therefore, the *CF* indicates the general performance of a specific type of turbine at a given location by combining local wind conditions and its inherent efficiency. It is a design parameter for a wind farm related to its economic feasibility, usually taken as a constant during its whole lifetime. However, any reliable estimation of future changes can certainly contribute to a better feasibility assessment for any tentative project. *CF* provides a straightforward way (Eq. 1) of calculating the annual electricity production ($AEP_{wind}$) for a given *WT* such as the one selected for this study.

1 $$\begin{aligned} CF=\frac{AEP_{wind}}{P_R \cdot 365.25 \cdot 24} \end{aligned}$$

**Figure 1:**
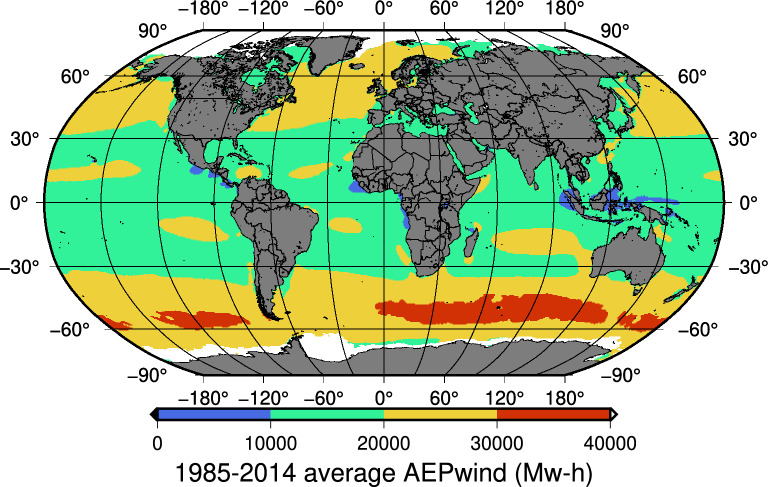
ERA5 1985–2014 average $AEP_{wind}$ for the selected 5 MW device. Map created by authors using GMT-6^29^.

The ECMWF ERA5 hourly reanalysis data^28^ between 1985 and 2014 were used to calculate current global CF values for wind and wave variables (In "Average annual wind energy production" section). The 1985-2014 $AEP_{wind}$ values for the reference turbine were computed, thus providing a general snapshot of its hypothetical current average annual energy production (Fig. 1).

Robust Theil-Sen trends^30,31^ of $AEP_{wind}$ have been calculated using global WWIII monthly anomalies for the 2015-2100 period. Both ERA5 and WWIII data are projected onto the same 0.5$^{\circ }$x0.5$^{\circ }$ geographical grid. Global ocean bathymetry has been downloaded from NOAA^32^ and then reloaded onto the same 0.5$^{\circ }$x0.5$^{\circ }$ arrangement. Marine areas located less than 200 km offshore, with depths below 1000 m and average wind speeds at a hub height (90 m) of more than 5 m/s have been selected as candidate locations for *WT*s.

Several studies propose different hub height wind speed values ranging between 4 m/s^33^ and 7 m/s^34,35^ for a wind farm to record a profitable Levelized Cost of Electricity (*LCOE*).

**Figure 2:**
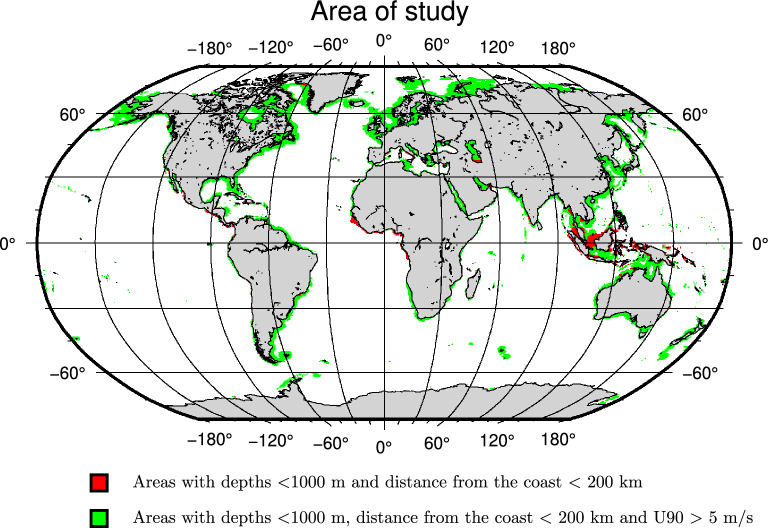
Coastal areas analysed. Map created by authors using GMT-6^29^.

For a global study such as this, a somewhat conservative common value of 5 m/s has been adopted as the threshold for identifying possible coastal locations worldwide that may have an economically positive profile. It must be highlighted that either *LCOE* or other economical indicators like the net present value or the internal rate of return, are basically dependent on the energy produced.

These constrains reflect current economic and technological boundaries^35^ but may well expand over the coming decades. Other possible limitations such as the density of grid connections, wildlife conservation areas^23^, communication towers,^5^, or even disputed territorial waters will not be considered here. As a result, the area analysis covers a total of 13409 candidate grid points corresponding to coastal regions where both bottom-fixed and floating wind farms could be technically and economically feasible (Fig. 2). Trends in $AEP_{wind}$ driven by climate variability in wind until 2100 have also been calculated at the selected locations (In "Calculation of wind energy production trends" section).

### Offshore wave energy: selected device, current energy production and global trends

The authors have already used a specific wave energy converter (*WEC*)^36^ for similar studies^37^. For this device, the *WEC*’s absorbed electric power $P_{abs}$ in KW (Eq. 2) depends on the local significant wave height $H_s$ (m), mean wave period $T_z$ (s) and diameter of the device $D_b$ (m). The electricity generated by this device depends on its size. However, in order to make trends’ results comparable at a global level and following previous works by the authors^37^, the selected diameter $D_b$ has been taken as 2 m.

2 $$\begin{aligned} P_{abs}= 4.5 D_b^{2.4} H_s^{1.7} T_z ^{-0.9} \end{aligned}$$.

**Figure 3:**
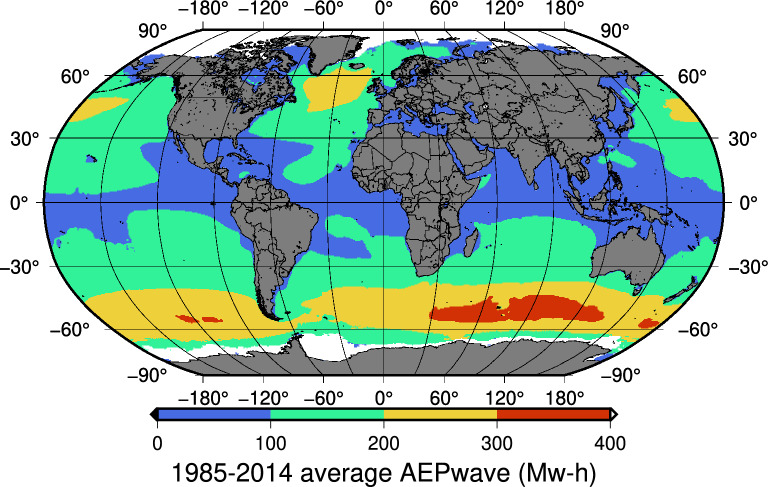
1985–2014 average $AEP_{wave}$ for the selected WEC. Map created by authors using GMT-6^29^.

Using the same 0.5$^{\circ }$x0.5$^{\circ }$ grid that was used for wind energy, the overall absorbed wave power under current-day conditions for the selected *WEC* was calculated using ERA5 hourly data of $H_s$ and $T_z$ corresponding to the 1985-2014 period. $P_{abs}$ was then used to calculate the overall annual energy production ($AEP_{wave}$) (Fig. 3).

For wave energy, Theil-Sen $AEP_{wave}$ yearly trends have been estimated subsequently from monthly $H_s$ and $T_z$ WWIII anomalies (see "Calculation of wind energy production trends" section), in coastal areas (15017 grid points) that meet the current constrains^35^.

## Results

In all cases, the trends for the two scenarios (SSP126 and SSP585) were evaluated at a 95% confidence level. The results are stated as a percentage per decade with respect to present-day values.

### Wind energy production trends

**Figure 4:**
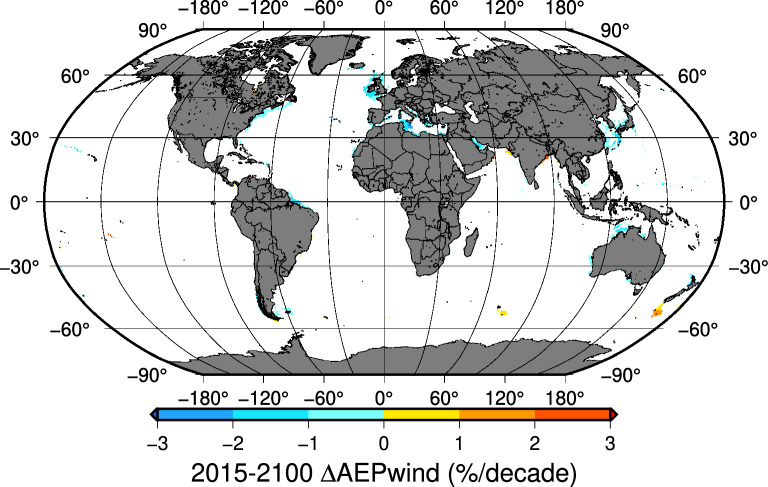
SSP585 scenario. Areas with significant $AEP_{wind}$ trends. 2015–2100. Map created by authors using GMT-6^29^.

In most of the candidate areas where wind farms can be installed, the results of $AEP_{wind}$ trends indicate that for both scenarios, no significant trend can be expected through 2100 (Table 1). In the case of SSP126, a fully static picture emerges until 2100. For the SSP585 scenario, the few areas with positive trends are concentrated in the Indian and Pacific Oceans at latitudes below 50$^{\circ }$S. The areas with negative trends represent only 12.2 % of all the grid points and include some coastal areas in the Mediterranean, Persian Gulf, Eastern Asia, the Atlantic seaboard of America and Northern Australia. The coastal areas of the British Islands also record negative trends for $AEP_{wind}$. However, in areas around the Baltic Sea or the English Channel, two regions with a high density of operating wind farms, no significant trends can be expected (Fig. 4).

**Table 1:** $AEP_{wind}$ global trends [2015–2100].

Scenario	$AEP_{wind}$ trends at 13409 gridpoints (GP)$^{1}$		
	No trend $^{2}$	Positive trend $^{2}$	Negative trend $^{2}$
SSP126	13267 GP [98.9%]	47 GP [0.4%]	95 GP [0.7%]
SSP585	11517 GP [85.9%]	254 GP [1.9%]	1638 GP [12.2%]

$^{1}$The $AEP_{wind}$ trends have been calculated at 13409 coastal gridpoints with depths smaller than 1000 m, less than 200 km offshore and hub height speed above 5 m/s

$^{2}$Theil-Sen trends at a 95% confidence level.

### Wave energy production trends

**Figure 5:**
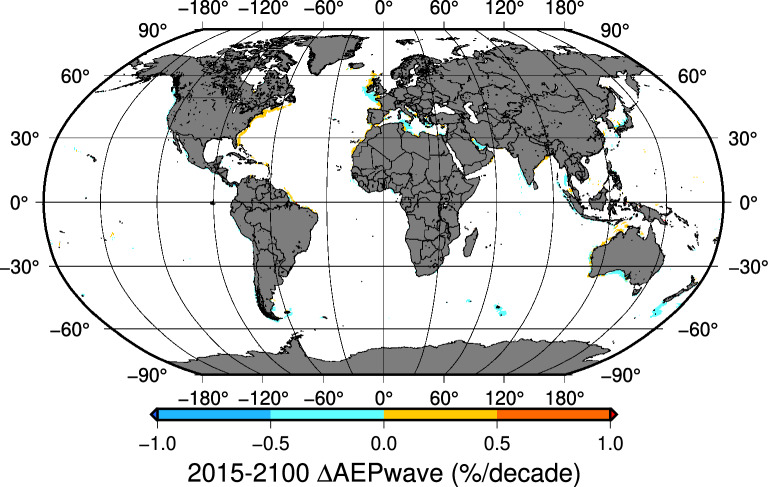
SSP585 scenario. Areas with significant $AEP_{wave}$ trends. 2015–2100. Map created by authors using GMT-6^29^.

Regarding $AEP_{wave}$ trends in the SSP585 scenario, no significant trend was detected in 84% of the areas analysed, and in the case of SSP126, only a tiny fraction of the candidate grid points (1.2%) recorded a significant trend (Table 2). Many grid points with significant trends in $AEP_{wind}$ and $AEP_{wave}$ in the SSP585 scenario tend to cluster around the same areas (Fig. 5) although in many cases, wind and wave energy record opposite trends.

**Table 2:** $AEP_{wave}$ global trends [2015–2100].

Scenario	$AEP_{wave}$ trends at 15017 GP$^{1}$		
	No trend $^{2}$	Positive trend $^{2}$	Negative trend $^{2}$
SSP126	14838 GP [98.8%]	126 GP [0.8%]	53 GP [0.4%]
SSP585	12596 GP [83.9%]	995 GP [6.6%]	1426 GP [9.5%]

$^{1}$The $AEP_{wave}$ trends have been calculated at 15017 coastal gridpoints with depths below 1000 m and less than 200 km offshore

$^{2}$Theil-Sen trends at a 95% confidence level.

## Discussion and conclusions

The SSP585 and SSP126 scenarios are two CMIP6 socio-economic boundary developments and their associated pathways of atmospheric greenhouse gas (*GHG*) concentrations. While *GHG* emissions are expected to fall by 2100 under the SSP126 scenario, SSP585 is the most pessimistic scenario^38^. The other scenarios record intermediate *GHG* emission patterns and adaptation policies. This paper’s main conclusion is that an almost static picture emerges for both boundary scenarios in most candidate locations for offshore renewable energy, specially in SSP126. In the case of SSP585, significant changes can be expected only in a reduced number of coastal areas, albeit with moderate values, mostly between 1% and 2% per decade. However, wind and wave energy trends as estimated from the devices selected in this paper, record opposite signs in many of those locations. This standardised approach makes results easier to compare through 2100 and this assessment could reasonably be extrapolated to other *WT* and *WEC* designs.

In the case of wave energy, its current stage of development renders it more difficult to gain a clear understanding of all the implications due to changes in $AEP_{wave}$ as calculated for the selected *WEC*. Mutriku (Spain) is the only wave farm in the world that has been continuously operating for more than 10 years^14^. It has proven to be both strongly resilient and adaptable to changes in the wave energy flux. The (*WEF*) regulation mechanism of its oscillating water column (*OWC*) technology mantain a constant average output, thus leveling off changes in the energy from waves^8^. Although some methodologies have been developed to compare different WECs^39,40^, in practical terms, it is difficult to predict how different wave converters based on other emerging technologies^41,42^ will perform. In the case of the *WEC* selected for this study, the expected trends in $H_s$ and $T_z$ for the coastal areas analysed record a constant energy production for most locations.

In the case of wind energy, *WT* designs in offshore facilities have evolved to converge towards three-blade models that differ mostly in size and in other minor details. This inherent similarity implies that the standardised results associated with the evolution of $AEP_{wind}$ corresponding to the device selected, can provide an accurate estimation of trends for any other current or future device. This is important because long-term changes in $AEP_{wind}$ may compromise or even improve to a certain extent the profitability of a wind farm.

Northern Europe, the Eastern seaboard of US, northern Japan, Italy, southwestern Australia and the Yellow Sea are the coastal areas concentrating most operational and projected new wind farms^43^. However, it is in these areas that negative $AEP_{wind}$ trends can be expected for the 2015-2100 period. Nevertheless, installation costs are currently falling and may even do so more in the future^5^. Although uncertainties remain in some key aspects such as the future of energy prices, the negative trend in electricity production in those areas even under the worst-case scenario (SSP585), is moderate and unlikely to involve major long-term changes or compromise economic feasibility through 2100.

In turn, areas such as the Arabian Sea or the Bay of Bengal, may record a boom in the wind energy industry due to their greater current and future potential. However, in these and other areas worldwide where offshore facilities could be deployed in the future, many fundamental elements such as roads and a local auxiliary industry are as-yet unavailable yet or have many limitations. All this constitutes an additional challenge, along with bottlenecks arising from licensing procedures, foundation technology and transnational power grid expansion.

Along these lines, remote territories in South America or the Southern Indian Ocean, such as the French Overseas Territories, Crozet and the Auckland Islands, have vast present and future potential for wind and wave energy (Figs. 1, 3). Thus, the series of methodologies and conditions identified by the authors^44^ and other researchers^45,46^ for different locations that are necessary for generating green hydrogen could also be met in these remote areas, thus making them the perfect location for its generation from wind and waves.

In general terms, the results presented here indicate that, regardless the final scenario reached by 2100, only moderate variations in the resources can be expected due to climate change and only in a few coastal areas. Thus, the impact of climate change on the economical and technological development of the offshore wind industry until 2100 can be reasonably predicted. For wave energy, a similar conclusion can be reached but greater caution is advisable because its future evolution also depends on ongoing technological developments. Other aspects such as detected changes in the frequency of extreme weather events or the hemispheric asymmetry in future wave power changes^47–49^ are beyond the scope of this study.

Although this and other uncertainties remain, the results shown here involve a reliable horizon for most ongoing feasibility studies, technological developments and investments in offshore facilities designed according to present-day conditions.

## Data and methods

ERA5^28^ hourly wind and wave data corresponding to this period, were downloaded from the European Centre for Medium Range Weather Forecast (*ECMWF*). This reference period has also been used to compute the average seasonal cycle from WWIII outputs and obtain the monthly anomalies for the 2015-2100 period. Robust global trends in wind and waves were then calculated until 2100.

### Data

The WWIII model has been embedded in two CMIP6 models (EC-Earth3 and ACCESS-CM2) for the SSP126 and SSP585 scenarios, and run with two different wind drag values identified as CDFAC1.0 and CFDC1.08^20^. The WWIII outputs include three-hourly global projections of $H_s$, $T_z$ and *WEF* from 1985 to 2100^18^ and the six available sets of WWIII outputs made available by CSIRO have been used here (Table 3).

**Table 3:** WWIII sets of outputs considered for this study.

Scenario	SSP585				SSP126	
CMIP6 model	EC-Earth3		ACCESS-CM2		EC-Earth3	ACCESS-CM2
Wind drag parameter	CDFAC1.0	CDFAC1.08	CDFAC1.0	CDFAC1.08	CDFAC1.08	CDFAC1.08

Monthly values were calculated for each set of outputs for the 1985-2014 period and anomalies were computed for the 2015-2100 period. Therefore, the seasonal components of the time series are removed, improving the detection of the trends^50^. Global Theil-Sen trends were then calculated for those anomalies. Finally, those trends were combined for both scenarios, considering an equal contribution from the two models and wind drag parameters thus providing a single trend for each ocean variable analysed and the two boundary scenarios (SSP126 and SSP585). Thus, the mean trend associated with all the elements in the ensemble is computed^51^. As a result, global trends for $U_{10}$ ($dU_{10}$/*dt*), $H_{s}$ ($dH_{s}$/*dt*) and $T_{z}$ ($dT_{z}$/*dt*) in their corresponding units per year were calculated for both scenarios. In a second stage, adopting the 1985-2014 period as a reference, trends for the 2015-2100 period have been expressed in percentage terms per decade.

### Average annual wind energy production

The present-day average $AEP_{wind}$ for the selected *WT* (Fig. 1) was estimated from the *CF* using 1985–2014 ERA5 data. The wind turbine’s rated power ($P_R$, 5MW), hub height (90m) wind speed ($U_{90}$), and diameter (*D*=123 m) were used for each ocean location to obtain the *CF* values with a widely used, semi-empirical expression^52^ adapted by authors (Eq. 3)

3 $$\begin{aligned} CF= 0.6 \left( 0.087{U_{90}} - \frac{P_{R}}{D^{2}} \right) \end{aligned}$$

The hub height wind speed ($U_{90}$) was calculated from the wind speed at 10 m ($U_{10}$) assuming a logarithmic profile^52^, with $U_{r}$ being the wind speed ratio (Eq. 4).

4 $$\begin{aligned} {U_{r}}=\frac{U_{90}}{U_{10}} = \frac{ \log (90/z_0) }{ \log (10/z_0)} \end{aligned}$$

where $z_{0}$ is the sea surface roughness which can be calculated^53^ from the significant wave height $H_s$ and wavelength *L* (Eq. 5)

5 $$\begin{aligned} z_0= 1200{H_{s}}\left( \frac{H_{s}}{\text {L}} \right) ^{4.5} \end{aligned}$$

In deep waters, the wavelength^54^ can be estimated from the mean wave period (Eq. 6)

6 $$\begin{aligned} L= \frac{g{T_{z}}^{2} }{2\pi } \end{aligned}$$

The *CF* values calculated in this way for the selected device are almost identical to other readily available estimations for similar *WT*s^55^ Finally, the overall $AEP_{wind}$ values (Fig. 1) were estimated from *CF* (Eq. 1).

### Calculation of wind energy production trends

Based on the above, equations their derivatives with time have been used to calculate the *CF* trends with respect to present-day conditions.

7 $$\begin{aligned} \frac{dL}{dt}= & {} \left( \frac{g}{\pi } \right) {T_{z}}\frac{dT_z}{dt} \end{aligned}$$

8 $$\begin{aligned} \frac{dz_{0}}{dt}= & {} 1200*5.5(H_{s}/L)^{4.5}\frac{dH_{s}}{dt} - 1200*4.5(H_{s}/L)^{5.5}\frac{dL}{dt} \end{aligned}$$

9 $$\begin{aligned} \frac{dU_{r}}{dt}= & {} \left( \frac{2log(3)}{ z_{0}log^{2}\left( 10/z_0 \right) }\right) \frac{dz_{0}}{dt} \end{aligned}$$

10 $$\begin{aligned} U_{90}= & {} U_{r}U_{10} =>\frac{dU_{90}}{dt} = \frac{dU_{r}}{dt}U_{10} + U_{r}\frac{dU_{10}}{dt} \end{aligned}$$

Following Eqs. (3) and (10), the *CF* trends in percentage terms per decade for each grid point with respect to present-day values have been calculated according to Eq. (11). These trends (Eq. 1) are the same for annual wind energy production.

11 $$\begin{aligned} \frac{dAEP_{wind}}{dt}[\%/decade]=\frac{dCF}{dt}=10*100\left( \frac{0.0087 }{CF} \right) \frac{ dU_{90}}{dt} \end{aligned}$$

### Average annual wave energy production

A floating body type model with a diameter of 2 m ($D_b=2m$) was adopted as the referential *WEC* for this study operating under deep water conditions^36^, with the absorbed wave power $P_{abs}$ in KW expressed as a function of $H_s$ and $T_p$ (Eq. 2).

Assuming a $P_{abs}$ at each grid point, the average annual wave energy production (Eq. 12) for the 1985-2014 period was calculated (Fig. 3).

12 $$\begin{aligned} AEP_{wave}[MW-h]= P_{abs}[KW] *365.25*24/1000 \end{aligned}$$.

### Calculation of wave energy production trends

The yearly trends in $P_{abs}$ can be derived from Eq. (13)

13 $$\begin{aligned} \frac{dP_{abs}}{dt}= 4.5 D_b^{2.4} \left( 1.7 H_s^{0.7} T_z ^{-0.9} \frac{dH_s}{dt} -0.9H_s^{1.7} T_z ^{-1.9}\frac{dT_z}{dt} \right) \end{aligned}$$

and when expressed in percentage terms per decade with respect to present-day values, at each coastal grid point, the calculation of the annual energy production trends is straightforward (Eq. 14)

14 $$\begin{aligned} {\frac{dAEP_{wave}}{dt}} [\%/decade]=10\frac{100}{P_{abs}} \frac{dP_{abs}}{dt} \end{aligned}$$.

## Data Availability

The datasets analysed during the current study are available in the CSIRO Data Access Portal repository, https://data.csiro.au/collection/csiro:53176.
